# A Chironomid Record of Early-Middle Holocene Environmental Evolution in the Darhad Basin, Northern Mongolia

**DOI:** 10.3390/insects13050461

**Published:** 2022-05-13

**Authors:** Zhenyu Ni, Enlou Zhang, Sangheon Yi, Weiwei Sun, Xianqiang Meng, Dongliang Ning, Jin Cheul Kim

**Affiliations:** 1 State Key Laboratory of Lake Science and Environment, Nanjing Institute of Geography and Limnology, Chinese Academy of Sciences, Nanjing 210008, China; zyni@niglas.ac.cn (Z.N.); wwsun@niglas.ac.cn (W.S.); xqmeng@niglas.ac.cn (X.M.); 2 Geological Research Center, Korea Institute of Geoscience and Mineral Resources, Daejeon 34132, Korea; shyi@kigam.re.kr (S.Y.); kjc76@kigam.re.kr (J.C.K.); 3 Department of Petroleum Resources Engineering, Korea University of Science and Technology, Daejeon 34133, Korea; 4 School of Geography Sciences, Nantong University, Nantong 226007, China; ningdl@ntu.edu.cn

**Keywords:** chironomid subfossil, functional traits, paleoenvironmental reconstruction, Westerlies, Central Asia

## Abstract

**Simple Summary:**

The Holocene humidity evolution presents spatial heterogeneity in the Mongolian Plateau where multiple circulation systems converged. Inconsistent records of regional environmental evolution affect accurate assessment of lake ecosystems. Chironomid larvae are one of the most abundant benthic invertebrates in lakes. The taxa composition of their functional groups is largely determined by aquatic habitats. Therefore, analyzing the changes of their functional traits can effectively indicate the hydrological dynamics in the past. In this study, we analyzed the early-middle Holocene (9.0–4.5 cal kyr BP) subfossil chironomid assemblages of a sedimentary sequence from the Darhad Basin in northern Mongolia. At 9.0 cal kyr BP, the community structure suddenly changed from littoral taxa to sublittoral/profundal taxa, reflecting an environmental transition from a river or shallow lake condition to a deep lake environment, which lasted until 4.5 cal kyr BP. Those hydrological patterns are consistent with the humidity evolution in the Westerlies dominated region, except that the onset of wetness occurred one thousand years earlier when comparing our results with previous ones, which may be related to the melting of regional glaciers and permafrost caused by increased summer solar insolation.

**Abstract:**

Under the influence of various circulation systems, the Holocene humidity conditions on the Mongolian Plateau are spatially heterogeneous and the underlying mechanism is still ambiguous. The complexity of climate change may affect the accuracy of assessing lake ecosystem evolution. In this study, based on the precise chronology, a chironomid assemblage sequence from the Darhad Basin in northern Mongolia is analyzed to elucidate the hydroclimate variation during the early-middle Holocene. The results show that the chironomid communities changed suddenly from littoral taxa to sublittoral/profundal taxa at about 9 cal kyr BP, reflecting an environmental transition from a river or shallow lake condition to a deep lake environment. Thereafter, most parts of the paleolake remained at a relatively high level until 4.5 cal kyr BP. This hydrological pattern resembles the typical humidity variations in the Westerlies affected regions, except that the onset of wetter conditions occurred one thousand years earlier as reflected in our results. The melting of glaciers and permafrost in the basin resulting from the early increased summer solar insolation could be a feasible explanation for these time advances.

## 1. Introduction

The lakes on the Mongolian Plateau play an important role in sustaining the ecosystem stability there. However, influenced by the global warming and the increased human activity, they have been exposed to the problems of shrinking and salinization in recent decades [1]. Previous studies have illustrated that the natural and human-induced droughts can result in significant declines in lake biodiversity and ecosystem stability, which in turn endangered the lake ecosystem services [2,3,4,5]. The chironomids, one of the most common groups of benthic communities, can effectively reflect the changes of lake environment, including summer temperature, salinity, water depth, and nutrient conditions [6,7,8,9,10,11,12]. Their functional groups (mainly habitat adaptation and feeding traits) are especially sensitive to aquatic environment changes [13,14,15]. Therefore, understanding the long-term effects of climate change on chironomid communities will be of great help to better predict future lake ecosystem dynamics and to effectively promote lake ecological protection, especially in the Mongolian Plateau.

Climatologically, the Mongolian Plateau is particularly sensitive to climate change because it is linked to the high–low latitude climate system. It is jointly influenced by the low-latitude East Asian summer monsoon (EASM), the mid-latitude Westerlies, and the East Asian winter monsoon controlled by Siberian-Mongolian High [16]. Therefore, the lake system in this region is very sensitive to natural forcing factors. The Holocene climate change in this region has been reconstructed using various paleo-environmental proxies such as pollen, mineral composition, and geochemistry [17,18,19,20,21,22,23,24]. However, the spatial pattern of Holocene humidity changes on the Mongolian Plateau and its surrounding areas has been discussed for more than 20 years. The focuses of the debate are the maximum influence range of the EASM over the Mongolian Plateau and whether the evolutions of Holocene humidity in the Westerlies dominated region and EASM dominated region are synchronous [25,26,27,28,29,30]. For example, Herzschuh [25] proposed that the Westerlies and monsoon dominated regions experienced a similar humidity evolution history during the Holocene, while a completely different view suggested that the whole arid region of Central Asia experienced synchronous humidity changes that was roughly opposite to the EASM region [26]. However, some studies have shown that in the arid and semi-arid Mongolian plateau region, neither the Westerlies nor the monsoon climate pattern seem to be applicable [30]. This may be due to the fact that the Mongolian Plateau is located at the intersection of climate systems, and the mountain lakes are also affected by the development and melting of regional glaciers and permafrost. The complex environmental background, combined with the fuzzy chronological framework, hinders our understanding of environmental changes in this area.

The Darhad Basin, located in the northern part of the Mongolian Plateau, is almost completely filled by thick lacustrine sediments. However, because of the lack of an accurate chronological framework, those sediments have been poorly studied. In this study, we analyzed the chironomid subfossil assemblages in sedimentary core from an outcrop profile of the Hodon braided river in lake plain. Finally, the regional environmental changes during the early-middle Holocene were discussed by comparing our data with published ones. Our objectives are as follows: (1) understanding the evolution history of aquatic communities in the Darhad Paleolake based on accurate and high-resolution chronological series; (2) reconstructing the paleoenvironmental dynamics of early-middle Holocene; (3) revealing the lake ecosystem response to climate changes in the Darhad Basin.

## 2. Regional Setting

**Figure 1:**
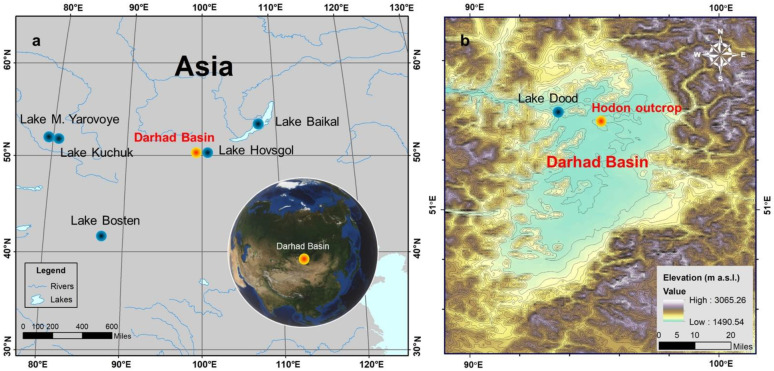
Location and setting. (**a**) Location of the Darhad Basin and other study sites mentioned. (**b**) Topographic map of the Darhad Basin and location of the Hodon outcrop and Lake Dood.

The Darhad Basin (50°38′ to 51°34′ N, 99°01′ to 100°00′ E) is located in northern Mongolia, near the Mongolia–Russia border (Figure 1a) [31]. It has a length of 110 km longitudinally and a width of 40 km zonally. This region is characterized by a typical continental climate, with the average annual precipitation ranging from 200 to 500 mm in the south and north, respectively. The mean January and July temperature is −30 °C and 15 °C, respectively, and the low temperature conditions led to the widespread distribution of permafrost in the basin [32,33]. Since the early Holocene, the surface of permafrost had been subjected to intense thermal erosion, which formed the thermokarst lakes in the lower part of the basin including the largest one in the northwest named Lake Dood (Figure 1b) [33]. Several large and medium rivers flow into the Darhad Basin, and the Shishhid River, the upstream of the Yenisei River, drains from the basin [34]. Resulting from the distinct climatic and geological conditions, three types of vegetation landscapes are formed in the region including the forest, forest-steppe, and steppe with very few forest coverage in the interior of the basin [35,36].

## 3. Materials and Methods

### 3.1. Coring and Chronology

A 13.2 m long sedimentary sequence was collected from the Hodon outcrop (1552 m a.s.l., Figure 1b) in the lacustrine plain in the north-central Darhad Basin in May 2010 [33]. The sediment core was cut longitudinally and then sectioned at 1 cm intervals in the laboratory. The chronology of the Hodon sequence was established based on the AMS ^14^C dating results of the eleven mollusk shells and five woods analyzed in ICA Laboratory, USA. All AMS ^14^C ages were calibrated to the calendar year (0 BP = 1950 AD) in the CALIB 7.1 program using the IntCal 13 calibration dataset [37]. The age–depth model was interpolated using a Bayesian model in the software package Bacon [38].

### 3.2. Laboratory Analysis

A total of 33 samples (approximately 2 g per wet sample) were collected from the Hodon sedimentary core at 35 cm intervals for chironomid subfossil analysis. The subfossil chironomid head capsule were prepared and analyzed using standard methods [39]. Sediment samples were flocculated in a 10% solution of KOH with a water bath at 70 °C for 20 min, and then washed through a 90 μm sieve. Chironomid head capsules were picked from the residual material using fine forceps under a stereo-zoom microscope at ×25 magnification. All head capsules were mounted on microscope slides using Hydromatrix^®^. Samples that produced less than 50 head capsules were excluded from further analysis [40]. The chironomid head capsules were identified mainly following the identification methods of Wiederholm [41] and Brooks [39]. According to habitat adaptability, chironomid community structure can be divided into littoral taxa and profundal taxa [39]. The feeding group of chironomid taxa is mainly composed of collector-gatherers, collector-filterers, predators, scrapers, and shredders [42,43].

### 3.3. Numerical Analyses

A percentage diagram of the chironomid taxa was produced using Tilia software, and the bio-stratigraphic zones were defined using stratigraphically constrained cluster analysis (CONISS) with Tilia [44]. Principal components analysis (PCA) was performed using CANOCO version 4.5 to investigate the dynamic evolution of chironomid communities [45]. The regime shift in chironomid communities were detected using the Sequential T-test Algorithm for Regime Shifts (STARS) [46,47]. A sequential analysis of mean values was conducted using the Student’s t-test to identify significant break-points and to detect possible regime shifts. The parameters of the significance level, cut-off length, and Huber weight were set as 0.01, 10, and 1, respectively. In addition, previously published biological and geochemical records of Lake Dood [20] were reanalyzed and processed.

## 4. Results

### 4.1. Core Description and Age Model

**Figure 2:**
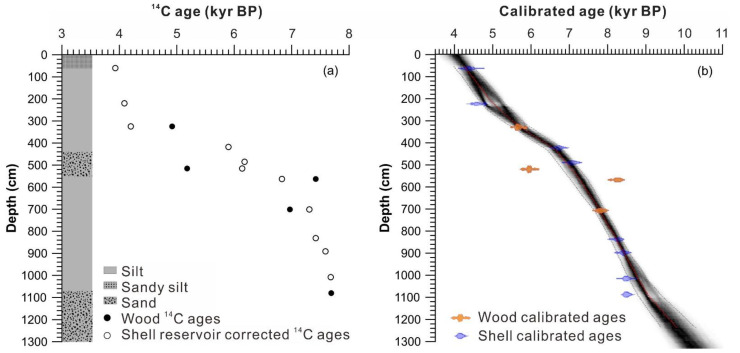
The lithology and age–depth model of the sedimentary core from Hodon outcrop [33]. (**a**) Radiocarbon ages of shells were corrected by 2450 years as reservoir effect and (**b**) all ages calibrated by Bacon age–depth model [38].

The 13.2 m Hodon sedimentary core is bounded at 4.1 m and shows two lacustrine sedimentary cycles, each consisting of silt and sand (Figure 2) [33]. The dating of mollusk shells and woods shows different trends, with a mean deviation of about 2.45 ka (Table 1). This difference might result from the uptake of old carbon in sediments by mollusks [48] and we assume that a constant reservoir age of 2.45 ka can be applied to correct all the shell dates before calibration. The final age–depth model results show that the Hodon core was deposited from 10.2 to 4 cal kyr BP, with the average sedimentation rate of about 2.1 m/ka. Detailed discussion about the chronological framework can be found in Krivonogov [33].

### 4.2. Chironomid Assemblages

**Figure 3:**
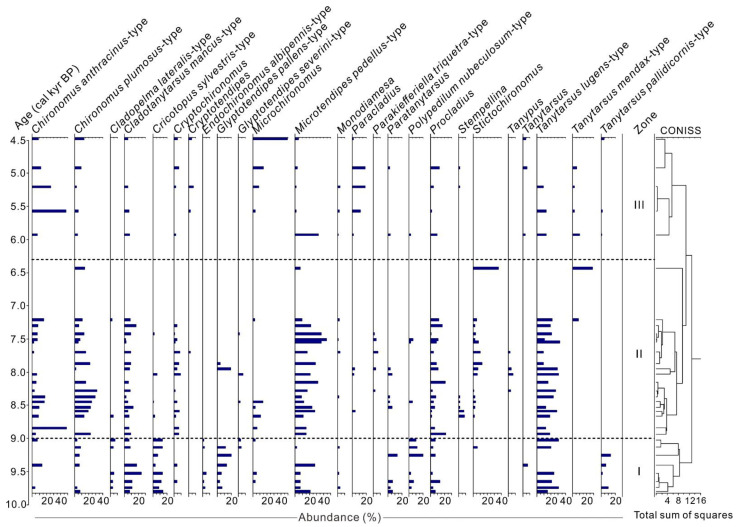
Relative taxa abundances (Hill’s N2 > 2) of chironomids in Hodon sedimentary core. The record is divided into three zones based on the constrained incremental sum-of-squares (CONISS) cluster analysis.

A total of 38 chironomid taxa were identified, including 28 taxa with Hill’s N2 > 2 (i.e., taxa that have a maximum abundance of more than 2% and/or occurred in more than two samples). The common littoral taxa are *Tanytarsus lugens-*type, *Cladotanytarsus mancus-*type, *Glyptotendipes pallens*-type, *Cricotopus sylvestris*-type and *Polypedilum nubeculosum*-type, etc. The normal profundal taxa are *Microtendipes pedellus-*type, *Chironomus plumosus-*type, *Chironomus anthracinus-*type, and *Stictochironomus*, etc. Five feeding traits are present in the assemblages, of which collector-gatherers and collector-filterers accounted for 53.8% (mean abundance) and 29.6%, respectively, while the shredders/scrapers and predators have a relatively low content of 9.2% and 7.4%, presented only in specific layers. The CONISS cluster analysis results divided the chironomid assemblages into three zones from bottom to top, which is remarkably consistent with the lithologic composition (Figure 3).

Zone I (1230–1050 cm, 9.7–9 cal kyr BP): The littoral taxa, including *Tanytarsus lugens-*type (mean abundance of 17.5%), *Cricotopus sylvestris-*type (10.7%), *Cladotanytarsus mancus-*type (10.5%), *Polypedilum nubeculosum-*type (7.7%), and *Glyptotendipes pallens-*type (7.7%) have the highest proportion compared to the entire sequence. In terms of feeding guilds, this period is characterized by a high abundance of shredders/scrapers. In contrast, the proportion of collector-gatherers is at the lowest level.

Zone II (1050–400 cm, 9–6.3 cal kyr BP): A regime shift in functional groups was identified with, notably, a near-disappearance of littoral taxa and a rapid increase of sublittoral and profundal taxa. *Microtendipes pedellus-*type (mean abundance of 24.9%) is the dominant taxon, and other common taxa include *Tanytarsus lugens-*type (17.6%), *Chironomus plumosus-*type (13.5%), *Chironomus anthracinus-*type (8.6%), as well as *Procladius* (7.8%), etc.

Zone III (400–100 cm, 6.3–4.5 cal kyr BP): profundal taxa are still the dominant type in this layer. The common taxa of *Chironomus anthracinus*-type (mean abundance of 17.0%) are *Microchironomus* (12.8%), *Paracladius* (8.9%), and *Tanytarsus mendax-*type (8.3%).

### 4.3. Ordination Analysis and Regime Shift Detection

**Figure 4:**
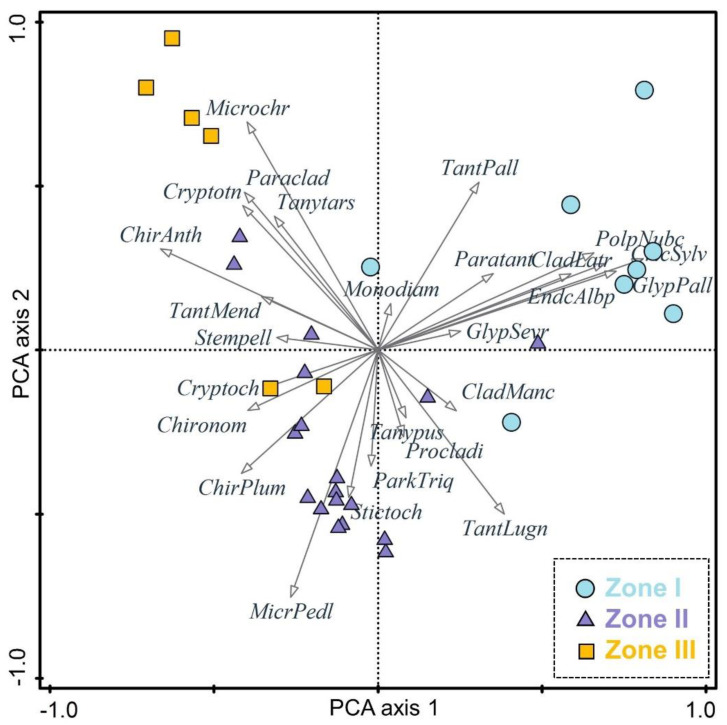
Principal component analysis of chironomid community in Hodon borehole.

The principal component analysis of the 28 taxa with Hill’s N2 > 2 shows that the first two principal component axes explained 39.05% of the total variability and the PCA 1 accounted for 20.8% (Figure 4). The littoral taxa, including *Glyptotendipes pallens*-type, *Cricotopus sylvestris*-type, *Polypedilum nubeculosum*-type, *Cladotanytarsus mancus*-type, and *Tanytarsus lugens*-type, are in the positive direction of the first axis. In the negative direction, the typical sublittoral and profundal taxa (*Microtendipes pedellus*-type, *Chironomus plumosus*-type, *Chironomus anthracinus*-type, and *Stictochironomus*) presented. According to the STARS analysis, the PCA 1 sample scores for chironomids indicates that a significant shift in the aquatic communities of Hodon sedimentary core occurred at ~9 cal kyr BP. In addition, previously published records of Lake Dood, including diatoms, total organic carbon, carbonate content, and inorganic carbon and oxygen isotopes [20], also indicate changes in the Darhad paleolake environment before and after the 9 ka event.

## 5. Discussion

### 5.1. Chironomid Community Evolution

The chironomid functional groups in the sediments of the Hodon outcrop are dominated by littoral taxa from 9.8 to 9 cal kyr BP. Most of them are shredders, scrapers, and miners whose diet is characterized by the living vascular plants, submerged wood, macro or colonial algae, or leaf litter [49] (pp. 136–168). They are often rare in central lake sediments but common in streams and fluctuating hydrologic littoral zones [50,51,52,53]. For instance, *Glyptotendipes pallens*-type and *Cricotopus sylvestris*-type are commonly found in sandy substrates in ponds connected by streams on the Mongolian plateau [54]. Plants in arid areas are distributed mainly in river valleys, where falling leaves, rotting wood, and macrophyte provide plenty of food for shredders. In neighboring northeast Russia, *Glyptotendipes pallens*-type is restricted to shallow areas in temperate lakes [11,55]. *Cricotopus* and *Polypedilum* taxa mostly live in the littoral zone where macrophyte flourishes in the arid lakes of Central Asia [56,57].

Around 9 cal kyr BP, there is a regime shift in chironomid community (Figure 3), with a rise in the proportion of sublittoral and profundal taxa (mainly collector-gatherers and filterers) and a rapid decline in littoral taxa. Collector-gatherers and filterers are the most common feeding mode and predominate in lakes where fine organic matter accumulates [49] (pp. 136–168). In Hodon sediments, the common taxa such as *Microtendipes pedellus*-type, *Chironomus plumosus*-type, and *Chironomus anthracinus*-type have typical collecting behaviors [13,49]. The abundances of *Chironomus plumosus*-type and *anthracinus*-type are positively correlated with water depth [56,58]. These hemoglobin chironomid taxa (mainly Chironomini) endure the low oxygen concentration of the deep water in lakes [49,59]. In addition, the proportion of predators (*Procladius*, *Cryptochironomus*) feeding on other small invertebrates increase significantly after 9 cal kyr BP, which implies a relatively high productivity in the lake caused by rising water levels. The transition from oligotrophic to mesotrophic lake state was also reflected in the rapid rise of *Microtendipes pedellus*-type abundance [60,61]. Therefore, the first axis scores of the principal component analysis of the chironomid community may indicate the paleolake water level dynamics in Darhad Basin.

### 5.2. The Aquatic Communities as Indicators of Regime Shift in Lake Environment

In large basins, the complex drainage system and surface material composition can result in spatial heterogeneity in the core sedimentary rate and grain size. By comparing the lithology results of multiple boreholes in Darhad Basin, the consistent continuous sedimentary sequence cannot be obtained [33]. It is obvious that the reconstruction of regional paleoenvironment by simple stratigraphic comparison may be defective. Bioindicators, like chironomids and diatoms in lakes, are thought to be effective indicators of environment changes, such as the fluctuations in water level [62,63,64]. The qualitatively inferred hydrological changes of Lake Bosten in Central Asia using the chironomids and diatoms are comparable [56]. Therefore, we can infer the regime shift of regional lake environment in the Darhad Basin by comparing the succession of aquatic communities in multiple boreholes.

**Figure 5:**
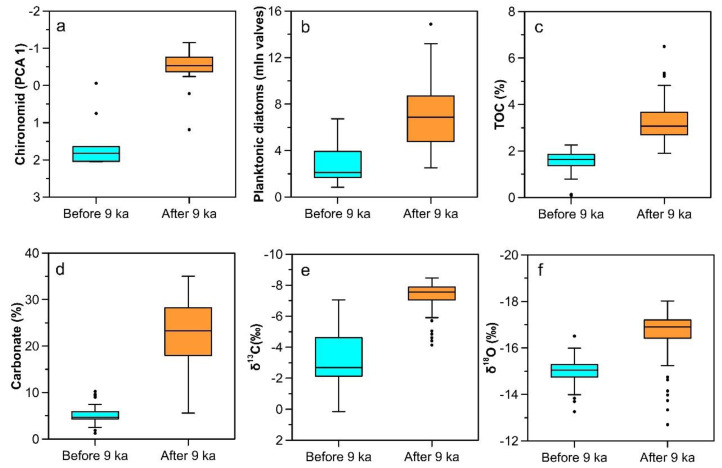
Changes of biological and geochemical indicators in sedimentary cores before and after 9 cal kyr BP, (**a**) PCA-1 scores of chironomid taxa in the Hodon outcrop, (**b**) planktonic diatoms, (**c**) TOC, (**d**) carbonate content, and (**e**,**f**) inorganic carbon and oxygen isotopes in Lake Dood [20].

Although the lithologic sequence of Lake Dood and Hodon outcrop was not reliably correlated due to unstable sedimentary processes and a relatively inaccurate chronological framework [33], the similar evolutionary trajectories of chironomid and diatom assemblages in the two cores can still obtain the key node of the regime shift in regional lake environment. The functional groups of chironomid assemblages in the Hodon core change significantly around 9 cal kyr BP with the disappearance of taxa in streams and coastal zones and a rapid increase in the proportion of deep-water taxa (Figure 3 and Figure 5a). At the same time, the dominant group of diatoms in Lake Dood also undergoes a transition from benthic to planktonic taxa, indicating a rapid rise in lake level and bio-productivity (Figure 5b,c) [20,65]. Therefore, chironomid and diatom communities in cores from different regions of the northern Darhad Basin jointly reflected a regime shift in the paleolake environment around 9.0 cal kyr BP. This phenomenon was confirmed by other multiple indicators. For example, a sudden increase in magnetic susceptibility at the 160–210 cm (after 9 cal kyr BP) layer of the sedimentary core from Lake Dood indicates an enhanced soil erosion caused by high river runoff [20]. A slightly higher C/N values also indicate small quantities of terrestrial carbon entrance into the lake after 9 cal kyr BP [20]. Besides, an increase of river runoff in the region might have carried a large amount of carbonate debris into Lake Dood (Figure 5d). The significant negative change of inorganic carbon and oxygen isotopes also confirmed the lake level rise at that time (Figure 5e,f) [20,66].

### 5.3. Regional Environmental Evolution and Possible Driving Mechanisms

The Darhad Basin is similar in structure to the neighboring Hovsgol Basin. Due to spatially uneven precipitation distribution, the latter is now a deep lake, while the former is filled with sediments [33]. The sediments of the Darhad Basin recorded several dammed lakes since Pliocene, and traces of paleolake can still be found during the last glacial–interglacial cycle [33,67,68]. Geological evidence and drift dating results from the Zyrianka glaciers indicate that the water depth of the glacial-dammed lake in the Darhad Basin was at least 140 m between 17 and 19 cal kyr BP, but most of the basin probably dried out after 10 cal kyr BP [67,68]. A few scattered small lakes, such as Lake Dood and Lake Targan, now occupy the northwestern part of the basin, but are insignificant in comparison to the basin. Nevertheless, the evolution of the aquatic ecosystems of these lakes and watershed wetlands can be used to infer the environmental changes of the Darhad Basin since the Holocene.

**Figure 6:**
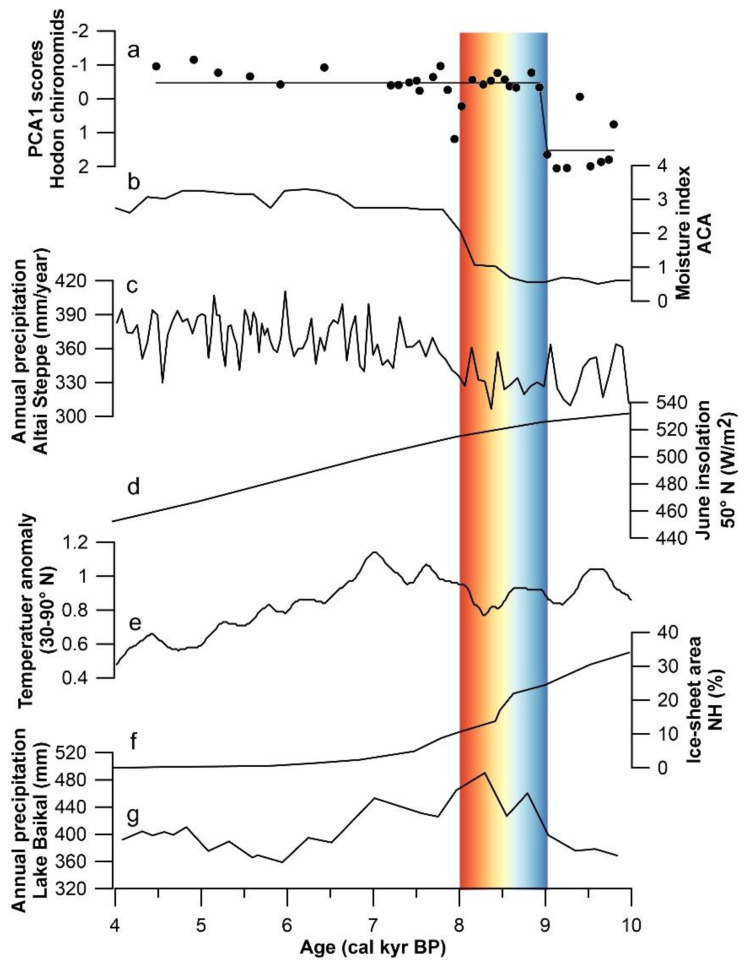
Comparison of (**a**) PCA axis 1 scores (dots) of chironomid assemblages (line: the regime shift detection using the STARS analysis) with other paleoclimatic records. (**b**) Humidity index in Arid Central Asia [26], (**c**) annual precipitation in the Altai Steppe [71], (**d**) summer solar insolation at 50° N [73], (**e**) temperature anomaly at 30–90° N [72], (**f**) Northern Hemisphere ice-sheet area [75], and (**g**) annual precipitation in Lake Baikal region [81].

Our results indicate that the succession of chironomid community indicates that the paleolake level in the Darhad Basin was low between 10.2 and 9 cal kyr BP, followed by a rapid rise thereafter (Figure 6a). The paleolake maintained a relatively high lake level, lasting for about 4500 years. Previous studies have shown that the water level of alpine lakes in the Northern Hemisphere is not only controlled by regional precipitation, but also affected by the melting of glaciers and permafrost in the basin, which leads to the inconsistency of lake dynamics in spatial distribution [29,30]. The annual mean meteorological data for Central Asia show that the moisture in this region is mainly transported from the North Atlantic and inland lakes along the mid-latitude Westerlies and cyclonic storms, respectively [69,70]. The environment evolution in this region during the early Holocene dry, middle Holocene humid and late Holocene relatively humid conditions, influenced by the intensity of Westerlies [26,28] (Figure 6b). The annual precipitation reconstruction results from the Altai Steppe using palynological data in Lake M. Yarovoye and Lake Kuchuk revealed that the Holocene humidity change history is consistent with the ‘Westerlies’ model [26], and precipitation began to rise around 8 cal kyr BP [71] (Figure 6c).

However, the high-water level in Darhad Basin appeared to be about 1000 years earlier than the high precipitation stage in Central Asia (Figure 6a). The driving factor may be the rise in temperature caused by strong solar insolation since the early Holocene [72,73,74] (Figure 6d,e). The rapid melting of glaciers and permafrost in the Darhad Basin increased runoff into the lake [75,76,77,78], which resulted in the rapid lake area expansion and water level rise [79,80] (Figure 6f). Similarly, the Holocene climate records reconstructed by pollen from Lake Baikal show that warm and humid conditions began to appear around 9.0 cal kyr BP [81] (Figure 6g). The humidity index based on pollen reconstruction from Lake Hovsgol recorded that the effective moisture increased at about 9.5 cal kyr BP in the region [21].

Since 4.5 cal kyr BP (the top 1 m depositions), the abundance of chironomid head capsules decreased significantly, and the amount has not reached the statistical threshold. This may indicate that the paleolake in the Darhad Basin shrunk and the location of Hodon outcrop had been far from the lake shore. The multiple proxies of the Lake Dood sedimentary sequence also recorded a decline in lake level due to reduced runoff into the basin [20]. Meanwhile, the cooling of temperature with less solar summer insolation might have resurged the permafrost and pingo in the basin and weakened thermal erosion, which lead to the drying of the thermo-karst lakes [33,82].

## 6. Conclusions

We analyzed the chironomid subfossils from a precisely dated sedimentary sequence in the Darhad Basin in the northern Mongolian Plateau. The results show that the functional characteristics of the chironomid community can effectively imply ecosystem changes in the long term. By comparing the chironomid records with diatom community evolution in the adjacent Lake Dood, it was found that the aquatic environment of the paleolake experienced a regime shift at about 9 cal kyr BP, which was supported by various physical and chemical indicators. During the 9–4.5 cal kyr BP, the area of the paleolake was larger and the water level was higher, which was consistent with the evolution of humidity in Central Asia dominated by Westerlies. However, there was a time lag of about a thousand years in initial wet conditions, which may be due to the melting of glaciers and thawing of permafrost in the basin which resulted from the increase of summer solar insolation during the early Holocene.

## Figures and Tables

**Table 1:** The AMS ^14^C dating results and calibrated ages of Hodon sedimentary core [33].

Depth (m)	Lab No.	Material	^14^C Age (yr BP)	Reservoir Eliminated Age (yr BP)	Calibrated Age (yr BP)
0.6	ICa100033	Shell	6380 ± 50	3930 ± 50	4380
2.2	ICa100034	Shell	6540 ± 60	4090 ± 60	4580
3.25	IWd100391	Wood	4920 ± 50	4920 ± 50	5670
3.25	ICa100035	Shell	6650 ± 50	4200 ± 50	4710
4.18	ICa100036	Shell	8350 ± 60	5900 ± 60	6730
4.85	ICa100037	Shell	8630 ± 60	6180 ± 60	7040
5.15	IWd100392	Wood	5180 ± 50	5180 ± 50	5940
5.15	ICa100038	Shell	8590 ± 60	6140 ± 60	7010
5.63	IWd100393	Wood	7420 ± 60	7420 ± 60	8270
5.63	ICa100039	Shell	9280 ± 60	6830 ± 60	7690
7.01	IWd100394	Wood	6970 ± 60	6970 ± 60	7810
7.01	ICa100040	Shell	9760 ± 70	7310 ± 70	8100
8.31	ICa100041	Shell	9870 ± 70	7420 ± 70	8210
8.91	ICa100042	Shell	10040 ± 70	7590 ± 70	8420
10.08	ICa100043	Shell	10130 ± 70	7680 ± 70	8490
10.8	IWd100395	Wood	7690 ± 60	7690 ± 60	8490

## Data Availability

Data are available upon request from the authors.
